# Client experiences using a new supervised consumption service in Sudbury, Ontario: A qualitative study

**DOI:** 10.1371/journal.pone.0292862

**Published:** 2023-10-16

**Authors:** Farihah Ali, Cayley Russell, Ashima Kaura, Peter Leslie, Ahmed M. Bayoumi, Shaun Hopkins, Samantha Wells

**Affiliations:** 1 Institute for Mental Health Policy Research, Centre for Addiction and Mental Health (CAMH), Toronto, Ontario, Canada; 2 Ontario Node, Canadian Research Initiative in Substance Misuse (CRISM), Toronto, Ontario, Canada; 3 Harm Reduction Worker, Co-chair Street Health Board of Directors, Founding Member of Toronto Overdose Prevention Society, Moss Park OPS and Toronto Harm Reduction Alliance, Toronto, Ontario, Canada; 4 Institute of Health Policy, Management and Evaluation (IHPME), Dalla Lana School of Public Health, University of Toronto, Toronto, Ontario, Canada; 5 MAP Centre for Urban Health Solutions, Li Ka Shing Knowledge Institute, St. Michael’s Hospital, Toronto, Ontario, Canada; 6 Department of Medicine, Temerty Faculty of Medicine, University of Toronto, Toronto, Ontario, Canada; 7 Division of General Internal Medicine, St. Michael’s Hospital, Toronto, Ontario, Canada; 8 The Works, Toronto Public Health, Toronto, Ontario, Canada; Laurentian University, CANADA

## Abstract

Overdoses are increasing in the province of Ontario, Canada, where northern communities such as Sudbury have witnessed disproportionately elevated rates, with opioid-related deaths double that of the provincial average. To address this issue, governments have implemented supervised consumption services (SCS) where people who use drugs (PWUD) can use their pre-obtained substances onsite under trained supervision. In September 2022, the city of Sudbury opened its first SCS, ‘The Spot’, but the site’s sustainability is contingent on demonstrating benefit to PWUD and the neighboring community. We undertook a qualitative study exploring experiences among clients who used the consumption service inside The Spot. In December 2022, clients of The Spot were invited to participate in a brief survey which collected socio-demographic information and substance use profiles, followed by an in-person semi-structured qualitative interview. Participant survey and interview data were combined with administrative site utilization data provided by site staff of all clients who accessed the consumption service from September 2022 to August 2023 to examine overall service utilization and uptake. Qualitative data were analyzed using iterative thematic analysis techniques, and results were informed by common responses to research questions. The responses were narratively presented. Administrative site utilization data highlighted a relatively stable increase in uptake and utilization of the site since its inception. A total of 20 clients participated in the survey and semi-structured interviews. Participants described the importance of the site in preventing and responding to overdoses, providing a safe and comfortable environment to consume their drugs, and decreasing public drug use, which they suggested may potentially reduce stigmatization in the community. However, clients also suggested challenges, including issues regarding site operational policies that hindered consumption room utilization. Service suggestions made by clients to improve site utilization include the addition of inhalation services, relocating the site to a location in downtown Sudbury where PWUD commonly congregate, and extending operational hours. Positive impacts and recommendations can be drawn on and considered by other northern or rural communities interested in implementing similar harm reduction services.

## Introduction

Substance use and related harms have sharply increased across Canada over the past decade, and especially following the onset of the COVID-19 pandemic [1]. For example, since January 2016, there have been 36,442 accidental opioid toxicity deaths in Canada, and the average number of deaths per day has doubled between 2019 (prior to the COVID-19 pandemic), and 2022. Moreover, more than half (56%) of the opioid deaths in 2022 also involved a stimulant [1]. Available data suggest that there have also been over 13,000 apparent stimulant toxicity deaths between 2018 to 2022, with many involving cocaine (64%) and methamphetamine (53%) [1]. Several factors are contributing to the overdose crisis, including increases in the prevalence of opioid use and opioid use disorders, increases in risky drug use, such as using drugs while alone, fentanyl-laced stimulants, and increases in the toxicity of the illicit drug supply [1]. In particular, the province of Ontario has witnessed the highest number of opioid and stimulant overdose deaths in Canada, with 2,501 and 1,811 occurring during 2022 respectively. However, within Ontario, overdose deaths are not evenly distributed, and there have been stark increases in opioid-related overdoses in northern and rural areas since the COVID-19 pandemic began. For example, Greater Sudbury, which is the largest city in Northern Ontario, with a population of approximately 166,004, experienced an opioid-related mortality rate of 44.6 per 100,000 individuals in 2021, which was more than double the Ontario provincial rate of 19.6 per 100,000 [2, 3]. Data from the Greater Sudbury Community Drug Strategy (CDS) further highlights a drastic increase in opioid-related harms. For instance, paramedic services responded to 896 suspected opioid-related incidents in 2021, which was almost double that of 2019 (n = 468) [3, 4]. Additionally, there were 636 visits to emergency departments for confirmed opioid overdoses, representing a rate of 309.5 per 100,000 people, which is substantially higher than the rate for all of Ontario (115.4 per 100,000) [4]. Almost half of opioid deaths in Sudbury in 2022 involved cocaine and a third involved methamphetamines [5].

To address this ongoing crisis, governments across Canada and within Ontario have implemented several harm reduction measures such as supervised consumption services (SCS), with thirty-eight sites operational in Canada as of 2023, twenty-six of which are located in Ontario, and two of which are located in Northern Ontario (i.e., Thunder Bay and now Sudbury) [6]. SCS are evidence-based services that provide space for people who use drugs (PWUD) to safely use substances, typically under the supervision of trained healthcare providers or public health professionals who can also provide referrals to treatment services [7]. SCS allow PWUD to use pre-obtained drugs by way of injection, intranasal, and/or oral consumption [7]. SCS improve overall individual and public health outcomes by mitigating risky substance use behaviours such as sharing or reusing needles, improving access to other health and social services, and reducing the risk of hospitalization and death due to overdose [7, 8].

Although the literature on the benefits of SCS is well established and demonstrates many benefits for PWUD and their communities, these services are less available and accessible in northern and rural jurisdictions in Ontario despite the disproportionately elevated overdose rates in many of these communities [3, 4]. Historically, SCS application requirements have been administratively demanding, making it difficult for smaller communities with fewer resources to be granted approval compared to larger urban settings. In many smaller communities, stigmatization of PWUD is prevalent and contributes to difficulties securing community ‘buy-in’ for SCS, especially considering community consultations are a federal and provincial application requirement. This often results in unsuccessful applications and subsequent limited uptake [8]. For instance, smaller communities are more likely to encounter challenges with meeting Ontario provincial SCS requirements related to the establishment of permanent sites, including location restrictions, rigorous evaluations, the inclusion of on-site treatment services, and addressing community concerns on an ongoing basis, which have all been identified as barriers to operationalization [9, 10].

The city of Sudbury, Ontario, is one such example of a community that has faced many challenges in their efforts to apply for a permanent SCS, underscoring the difficulties some Northern Ontario communities have endured in implementing a SCS. To meet application requirements and corresponding permanent site approval, the CDS undertook a needs assessment between 2019–2020 which consisted of a survey with 190 people who inject drugs (PWID), an online survey with 2,251 community members, focus groups with 52 community partners and stakeholders, and secondary data analysis [11]. The assessment noted that more than half (53.7%) of PWID surveyed reported experiencing an overdose, 58.8% of whom had experienced one or more overdoses in the last six months. Regarding risky substance use behaviors, 71.1% of PWID reported re-using their own needles, 32.6% reported using a needle previously used by someone else, 36.3% reported they had trouble obtaining enough sterile needles to suit their needs, 83.2% reported having injected alone, and 89% reported that they would be willing to use a SCS, if one were to become available in their community. Regarding the distance PWID indicated they would travel to access an SCS, 28% suggested they would walk for up to 20 minutes in the summer to access a SCS, 20% would walk for up to 30 minutes, while 19% would walk no more than ten minutes and 18% would walk no more than five minutes [11]. These results underscored the need for a SCS in the community, yet simultaneously uncovered community member and business owner apprehension, stigmatization, and not-in-my-backyard (NIMBY) sentiments in which residents typically supported the need for the service as long as the location of the service was far away [12, 13].

Following the needs assessment and the subsequent submission of a SCS application, in May 2022, Sudbury was granted a time-limited federal exemption from Health Canada to operate the community’s first federally sanctioned SCS. The temporary site, also known as ‘The Spot,’ opened on September 28, 2022, and provides an array of services such as distribution of harm reduction supplies, consumption services, and referrals to other social and health services. The site is run by the Réseau ACCESS Network, a non-profit, community organization committed to holistic and comprehensive approaches in promoting wellness, harm and risk reduction, and education, which operates in a different location than The Spot [14]. Although The Spot was federally approved, as of Fall 2023, it has not yet received provincial funding and is currently relying on $1.1 million in funding, provided over one year, from the Sudbury city council. Furthermore, the site and its location are currently temporary, with the site slated to close in December 2023, if long-term provincial funding is not granted.

Given the novelty of the service in the City of Sudbury after longstanding harms associated with drug use, and lack of certainty regarding the sustainability of the site, it is important to explore how the site is being experienced by PWUD within the community, including perceived benefits, challenges, and impacts. Gaining an understanding of such experiences and perceptions will be important to understand the site’s utility and ability to meet community needs. As such, we conducted a study to qualitatively examine client experiences of using the consumption service at The Spot through in-depth semi-structured interviews. This data were complemented by administrative site utilization data provided by The Spot staff which included overall uptake of the consumption service based on total number of visits and consumptions, as well self-reported substances consumed within the consumption room by clients. The results of the study can be used to demonstrate the benefits and challenges of the site as well as to inform decision-making practices regarding the sites’ sustainability and impact, including evidence of the community’s need for site permanency.

## Methods

The study involved a brief in-person socio-demographic survey followed by a qualitative semi-structured one-on-one interview. The survey collected basic socio-demographics including the age, gender, ethnicity, living situation, and substance use profiles of each participant. The qualitative semi-structured interview guide was developed in collaboration with the primary research team and peer advisors. The interview questions explored participants’ initial perceptions of the site, including any benefits, challenges, and barriers to accessing the site, as well as any impact it has had on, or will potentially have on, drug use patterns and risks of associated harms, and whether, or to what extent, the site is meeting the needs of PWUD. In addition, aggregate site utilization data between September 28, 2022 and August 31, 2023 were provided by the site staff which included monthly totals of the number of consumptions, visits, new clients, as well as the number and types of drugs consumed by clients who accessed the consumption service to contextualize overall consumption room utilization.

### Eligibility criteria

Clients were eligible if they were: aged 18 years or older, currently living in Sudbury, currently using illicit substances, fluent in English, and had used the consumption service at The Spot at least once. Clients who attended the site only for harm reduction supplies or did not speak English were excluded from the study.

### Recruitment

A sign-up sheet that included three days to participate in a scheduled interview (with 60-minute time slots) was circulated by staff members two weeks in advance of data collection. Staff assisted with recruitment by posting recruitment flyers at consumption booths and spreading the word to clients via word of mouth. Interested clients signed up at a specified time slot. In addition, the research team allocated two additional drop-in days where consumption room clients could participate on a first come first served basis as they accessed the consumption room, with no prior appointment needed.

### Data collection

The study was conducted between December 1 and 5, 2022 in a private room at The Spot, which allowed two trained members of the research team (FA and CR) to conduct the interviews. The research team collected written informed consent from participants prior to commencing the study. Clients were asked to participate in a survey lasting about five minutes whereby demographic characteristics and substance use profiles were captured via an online tool (Research Electronic Database Capture [REDCap]). A 30-minute, audio recorded, one-on-one qualitative semi-structured interview was subsequently conducted, which asked clients about their experiences and perceptions of the site. Study participants were assigned a unique code to maintain their confidentiality. To compensate clients for their time and participation, $30 cash honoraria was provided upon completion of the study. The study was approved by the Centre for Addiction and Mental Health (CAMH) Research Ethics Board (#087/2022). From September 2022 to August 31, 2023, aggregate site utilization data were recorded monthly on Excel documents by staff at the site and shared with the research team via a Microsoft word document that provided monthly breakdowns of the total number of consumptions, visits, new clients, as well as the number and types of drugs consumed by clients who accessed the consumption service. Number of overdoses that were reversed at the site were also included in the data (including non-fatal and fatal, overdoses requiring naloxone, or overdoses requiring either emergency medical services [EMS] or other medical emergencies). The site utilization data were collected by staff and clients via an intake form which was completed with every client prior to entering the consumption room (regardless of whether it was their first visit or not). Data on the type of drug clients intend on consuming in the room, based on what they believe they have purchased (i.e., a question asking, ‘what substance will be used today?’) was also collected.

### Data analysis

All quantitative survey data (i.e., socio-demographics and substance use profiles) were exported into Excel from REDCap. For both the quantitative survey data and the aggregate monthly site utilization data provided by staff, basic descriptive statistics were applied.

All qualitative data audio files were uploaded onto a secure network, and later transcribed verbatim via a third-party company, with all identifying information removed. The transcripts were reviewed for accuracy, and imported into qualitative software (NVivo, v12). An initial codebook of themes was prepared based on the interview guide, research questions, and preliminary analyses (e.g., perspectives on benefits of the site, perspectives on challenges of the site, and perspectives on improvements for the site). The transcripts were reviewed by two members of the research team (FA and CR) who identified and coded common themes. The codebook was subject to further development and revision based on ongoing analyses and discussion of emergent themes among members of the research team. All qualitative data were analyzed using iterative descriptive thematic analysis techniques, informed by Braun & Clarke’s (2006) six phases of thematic analyses [15]. Themes were informed by common responses to the research questions. Any coding discrepancies were discussed among team members and agreed upon. The responses were collapsed into overarching categories and narratively reported.

## Results

The first section of the results reports overall usage of the consumption room, based on administrative aggregate site utilization data retrieved from The Spot. This was used to identify overall uptake of the consumption room in addition to client substance use profiles. The second section of the results highlights participant data from the survey, documenting their socio-demographic characteristics and substance use profiles. This is followed by the qualitative findings.

### Site utilization data

**Fig 1:**
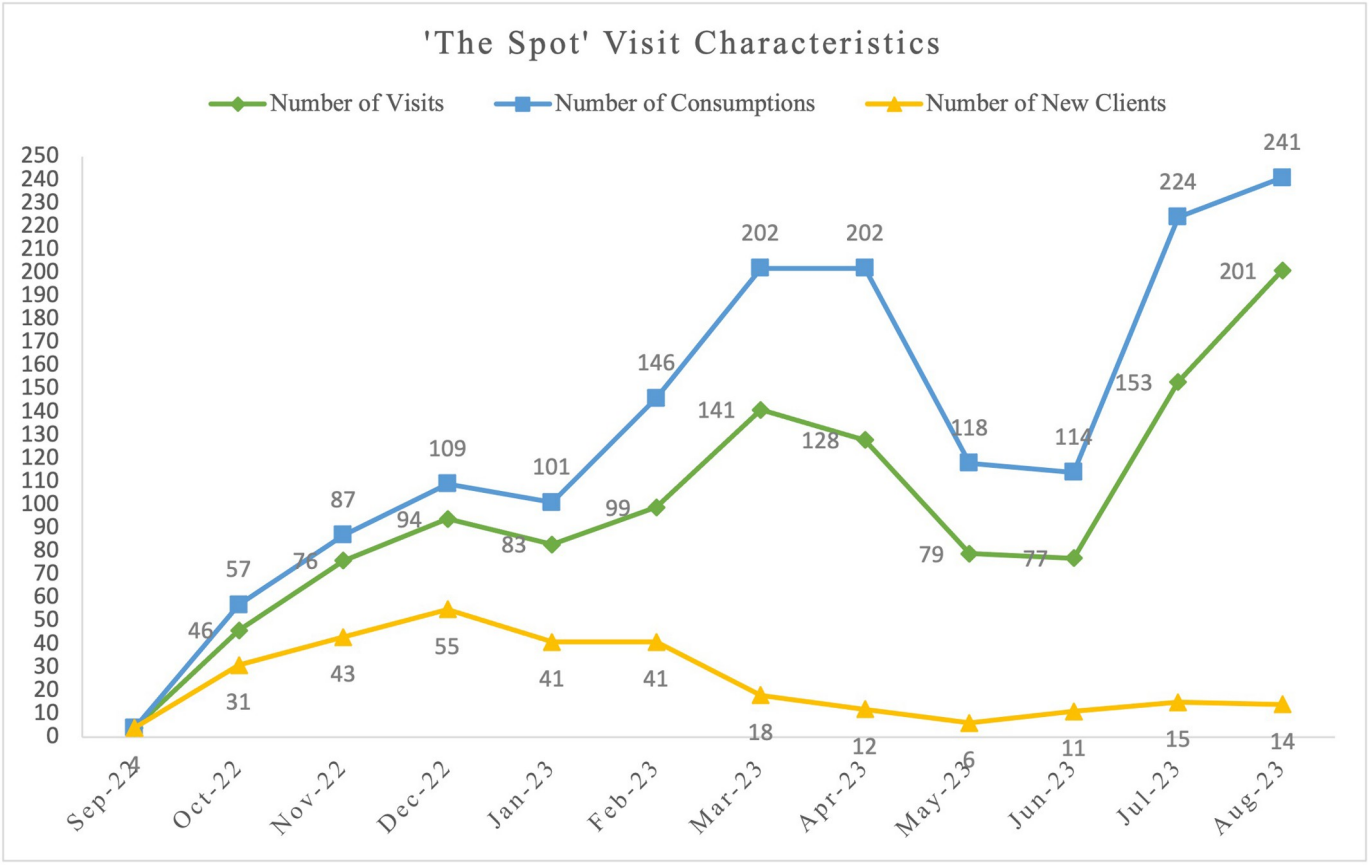
The Spot consumptions, unique clients, and visits, based on site utilization data collected between September 28, 2022, and August 31, 2023.

Between September 28, 2022, and August 31, 2023, a total of 470 unique clients accessed The Spot, representing 1,181 total visits, and 1,605 total consumptions (see Fig 1). All site visits are counted once, irrespective of whether the client has visited the site before or how many times a client may access the site in a day. Each individual consumption is counted as a unique consumption, whether or not it is the same individual utilizing the consumption room multiple times a day or using the service once but consuming multiple times in one visit. This can lead to instances where the number of consumptions exceed the number of visits.

Of the drugs reported, fentanyl was the most commonly self-reported injected drug with 1,005 consumptions, followed by speedballs (i.e., a mixture of opioids, now most commonly fentanyl and stimulants, typically either cocaine or methamphetamine) (n = 286 consumptions), and methamphetamine (n = 111 consumptions). The Spot has reversed a total of 20 overdose events, nine of which required naloxone, the remaining seven required oxygen only as the intervention. No overdose required EMS.

### Socio-demographic characteristics of participants

A total of 20 clients participated in the study (**Table 1**). The average age of participants was 37, with the majority aged 31–50 (n = 12; 60%). Nearly three-fourths identified as men (n = 14; 70%), and half (n = 10; 50%) identified as Indigenous. Almost half (n = 9; 45%) reported experiencing homelessness.

**Table 1:** Participant demographic characteristics.

Demographic Characteristics (N = 20)	Frequency (n = 20)	Percentage (%)
Age (years, mean ±SD)	37.2 ±9.5	
Age Groups		
*18–30*	6	30
*31–50*	12	60
*≥* 51	2	10
Gender		
*Man*	14	70
*Woman*	6	30
Ethnicity		
*White*	10	50
*Indigenous*	10	50
Living Situation		
*Stably Housed*	6	30
*Unstably Housed*	5	25
*Homeless*	9	45
Experienced overdose since site opened	0	0
Number of times accessing the site		
*Less than five*	16	80
*More than five*	4	20

Regarding substance use characteristics among participants, fentanyl was the most commonly self-reported substance used within the past 30 days (n = 19; 96%), followed by crack cocaine (n = 17; 85%), and methamphetamine (n = 15; 75%). In terms of routes of consumption, the most common route of illicit opioid consumption was injection (n = 18; 95%) followed by inhalation (n = 14; 78%). Of the 17 participants who used crack cocaine, the most common route of consumption was inhalation (n = 16; 94%) followed by injection (n = 4; 24%). Of the 15 people who used methamphetamine, injection was the most common route of consumption (n = 11;73%), followed by inhalation (n = 9; 60%). See **Table 2** for participant substance use characteristics.

**Table 2:** Participant substance use characteristics.

Substance Use Characteristics (N = 20)	Frequency (N)	Percentage (%)
Substances used in the past 30 days*		
**Powder cocaine**	9	45
*Route of Consumption*		
*Injection*	7	78
*Inhalation*	4	44
*Nasal*	2	22
**Crack cocaine**	17	85
*Route of Consumption*		
*Injection*	4	24
*Inhalation*	16	94
*Nasal*	1	6
**Methamphetamine**	15	75
*Route of Consumption*		
*Injection*	11	73
*Inhalation*	9	60
*Nasal*	1	7
**Illicit Opioids (fentanyl)**	19	96
*Route of Consumption*		
*Injection*	18	95
*Inhalation*	14	78
**Prescription Opioids**	8	40
*Route of Consumption*		
*Injection*	3	36
*Nasal*	1	13
*Oral*	4	50
**Stimulant Opioids**	10	50
*Route of Consumption*		
*Injection*	10	100
*Inhalation*	4	40
**Other**	15	75

* Categories are not mutually exclusive. The frequency of route of consumption is calculated out of the denominator for each respective substance, not the total number of participants.

### Qualitative findings

The findings from the study are presented narratively below and organized into the following thematic categories: 1) Perceived impacts on substance use, including perceptions of overdose risk and frequency of substance use; 2) Perceived impacts on stigma, including community-level stigma, and 3) Perceived operational impacts, including existing policies (e.g., inhalation services not provided, splitting/sharing substances not permitted), location, and hours of operation. The themes are illustrated with select quotes from participants, where appropriate.

#### Perceived impacts on substance use

*Perceptions of overdose risk*. The most common benefit of the site identified by participants related to increased feelings of safety when using the site to inject their substances. All participants stated that they felt safe using their substances in the consumption room where their use and any potential adverse effects were monitored by trained staff in proximity. This was particularly relevant for participants who expressed a fear of overdosing (n = 16; 80%) and suggested they felt at ease knowing that if they were to overdose, there would be trained staff present to intervene. For instance, when asked why they decided to come to The Spot, one participant described:

***“****For safety*. *Because I would just prefer to be somewhere where I’m safe and know If I do overdose*, *there’s someone there to help me… I’m here for that safety reason like I said*, *I don’t want to die*. *Bottom line*.*”*

Some participants specifically described the importance of having trained staff available on site when using their drugs as a means to prevent and respond to overdoses, as they indicated they could not trust their friends or peers to intervene in the event of an overdose. Some participants provided anecdotes of situations they had been in where the people they were using with had left them during an overdose: “*I almost died a couple times*, *and my friends I was with at the time just left me there*, *you know what I mean*? *They won’t let me die in here*.*”*

Another participant reiterated this sentiment, and suggested justifications for why people typically leave the scene when someone overdoses, including a fear of criminalization or because they did not want to ruin their own high, which would not happen at the site:

*“A lot less people aren’t going to lose their life because of [The Spot]*. *It makes me sick because I’ve been around so many people that have overdosed and everyone just leaves because they’re scared to get involved with the police and say “oh it’s a buzz kill*.*”*

Participants who disclosed that they typically used alone (n = 8; 40%) also reiterated the importance of being able to use under the watch of staff as an important overdose mitigation and lifesaving strategy: *“Just to be in a safe environment*, *and if I’m using by myself and I come here at least I don’t have to worry about overdosing*.*”*

*Frequency of substance use*. Although the site had only been open a short while, participants discussed the positive impacts the site could potentially have on the frequency of their substance use long-term. Half (n = 10; 50%) of participants suggested that if they limited their drug use to only injecting when they are at The Spot, it could help them reduce their use:

*“It’d kind of help me slow down*. *Like if I chose to just do it here…[I would use less]… and if I wanted to just quit*, *that way I won’t go anywhere else like on the street or you know*, *like my place of residence by myself*, *you know*? *[I would use the site to slow down and eventually quit]*.*”*

Participants speculated that the main reasons they might limit their use to only when they were able to access the site was due to increased feelings of safety when consuming there:

*“I will not inject anywhere else other than here*. *Not at a friend’s house*. *Not even with another person if they’re with me*. *I just won’t*. *I don’t feel comfortable…there’s many times when I won’t inject because they’re closed*.*”*

However, while some participants suggested The Spot could potentially support reductions in drug use, two participants indicated the opposite, and described the possibility for their use to increase due to the same feelings of safety when consuming at the site, and the assurance that they would be attended to in the event of an overdose.

#### Perceived impacts on stigma

The presence of stigma against drug use and PWUD within the Sudbury community was commonly discussed by participants. Participants suggested that the longstanding visibility of public consumption and subsequent paraphernalia littering had prompted negative community attitudes and perceptions towards PWUD and had reinforced stigma against them. These attitudes were heightened due to the implementation of The Spot, where some participants (n = 9; 45%) discussed how the site was not supported by many Sudbury residents and business owners—many of whom felt as though the site enabled drug use:

*“There’s a stigma attached to this place for sure*. *I personally don’t care*. *Well I can’t say that*. *I do care what people think*. *I think no matter where this building is there’s always gonna be negative people*. *Saying you’re enabling them*. *But it’s an epidemic*, *people are dying*, *they need places like this to keep people alive… I’ve had three people die over the weekend of overdoses*, *because they were using outside by themselves*.*”*

One of the main issues related to community-level stigma as described by participants was the increased visibility of public drug use. Participants discussed how public drug use had become entrenched and normalized, particularly within the downtown Sudbury area, and participants disclosed that they were frustrated and sad about this reality:

*“Five years ago you could walk around*, *there wasn’t needles everywhere*. *Now*, *it’s like every corner you go by there’s a needle*, *an uncapped needle on top of that*. *Even parks where kids play*, *I found a few of them sitting there*. *I’ll grab them and pick them up and put em in my pocket and go discard them because it’s not fair for a kid*. *Like what if they take off their shoes and run in the sand and get pricked by a needle and what*, *they get sick*, *they get AIDS*, *something like that*? *That’s not cool*.*”*

However, participants speculated that the presence of the site could potentially work to mitigate the visibility of public consumption, which could reduce community-level stigma towards them. Participants suggested that if the site worked to encourage people to only use there, where they can safely discard their used equipment and use away from the public’s gaze, that this could potentially alleviate community concerns:

*“It’s gotten to be too much of nothing for drug users to be walking around and probably nodding off and there’s a good chance they could be dead and [pedestrians] just walk right by*. *If they’re [at The Spot] it’s good for a lot of people*, *the people who don’t die*, *the people who don’t have to see it*, *kids*.*”*

Some participants elaborated on the site’s potential to not only reduce public consumption, but to also increase community safety via less littered paraphernalia and discarded needles. Participants stressed the potential for the site to be able to reduce the number of discarded needles, and that this could be a major benefit to the community:

*"What would they [Sudbury community] rather have*? *More people laying on the sidewalk or more needles downtown*? *Anyone who doesn’t want this [The Spot] here doesn’t get the benefits of it*. *You get more people sleeping in daylight*, *in front of business*, *shop owners having to clean needles out of their front every day*. *Even the closed-minded people have to get that*.*"*

However, just over a third of participants (n = 7; 35%) suggested that community-level stigma was so engrained within the community that the site would likely not make a difference:

*“[The stigma] has gotten so bad in this city*. *The people who don’t use are just like*, *oh*, *another one*. *You can learn ten thousand great things about me*, *but the second you learn I’m a junkie*, *that’s it*. *I don’t think we’ll ever get rid of that*. *There’s always gonna be a stigma attached to someone*.*”*

#### Perceived operational impacts

*Peer harm reduction staff involvement*. Among the positive attributes of the site that were discussed by participants, the presence of peer harm reduction workers with lived and living experience with drug use was noted as an invaluable asset. Participants emphasized that the presence of these workers provided them with comfort and suggested that this contributed to fostering a non-stigmatizing and non-judgmental environment. Knowing that staff had extensive knowledge about drugs made PWUD feel more comfortable as they could relate to them:

*“Well*, *the one guy*, *he’s been where I am*, *so he knows*. *I don’t know exactly where he’s been*, *but I know [he has lived experience]*. *Like hands-on*. *He’s given me advice on stuff*. *Changed my life talking to him and stuff*.*”*

For some participants, the presence of peer harm reduction workers was considered inspirational, as they saw themselves reflected in the workers and were motivated by the idea that people in their shoes could possibly stabilize and manage their drug use and attain employment in a field where they are knowledgeable and passionate:

*“It’s nice to see some of the local people here*. *They’ve got drugs*. *They’re working*. *They’re off the street*. *They look clean and fresh and they’re not using on site*, *I can tell*. *They’re holding it down*. *Kudos to them*. *To get up and go to work and hold it down all day*.*”*

*Lack of inhalation services*. A commonly discussed issue with the site was that inhalation services where clients could smoke their drugs were not offered or available and inhalation on site was not permitted. Most participants (n = 19; 95%) both injected and smoked their substances, some of whom identified a preference for inhalation (n = 4; 20%). As such, the majority of participants (n = 17; 85%) expressed the importance of inhalation services and suggested that the lack of inhalation services was a potential deterrent to accessing the site. Participants further noted confusion regarding the ability to inject but not smoke their substances, and given the dangers of inhaling substances, did not understand the rationale behind this policy:

*“If you’re allowed to shoot up here*, *you should be allowed to smoke up*. *I understand the cigarette part*, *but having a separate room*, *well ventilated and let the people smoke their fentanyl*. *You can come up here and shoot crystal meth but you can’t smoke it*? *Same with the down*? *You can’t smoke the down but you can shoot it*? *You should allow it because you can just die just as easy and no one’s there to help you*. *I wonder why they don’t allow it*. *That’s a bullshit policy*.*”*

Participants further expressed that because the site does not offer inhalation services, The Spot is therefore not able to support a large percentage of the population of PWUD considering many inhale their substances. Furthermore, some participants described how the risks associated with inhalation are just as high as with injection, and suggested that the site therefore does not offer harm reduction and safety for the entire PWUD community:

*“I’d say half or more of the people smoke [their drugs]*. *So*, *you have to take that into consideration too*, *and how many people have died from just smoking*?*”*

Despite some participants indicating the risks associated with inhalation, many believed that smoking their substances was a safer route of administration compared to injecting. For instance, some participants (n = 4; 20%) who typically consumed their drugs via both injection and inhalation mentioned that they would first smoke their substance before injecting as a harm reduction measure to test the toxicity or to see how strong it was. These participants believed that once they tested their substances via inhalation, they would be able to better determine whether it would be safe to inject: *“I smoke a bit as a risk mitigation strategy*, *and I feel it’s safer than injecting*. *It takes a while to feel the effects of it*.*”*

Participants further elaborated that if the site offered a space to inhale, they would use the site more often, including to practice what they considered harm reduction through testing their substances via inhalation prior to injecting:

*“There’s also a far lower chance of overdosing from smoking*, *but there still is*. *A lot of people think you can’t overdose if you smoke it…I think a lot more people would use here [if the site offered inhalation services] because a lot of people do both at the same time…Like I said*, *I smoke my down before I shoot it*, *just in case it’s too strong*, *right*? *[I would come here to do that test]*. *With fentanyl*, *I have watched people with a huge tolerance who do this shit every day*, *do one puff*, *not even exhale yet*, *and they’re dropped*. *One puff*. *Now that fentanyl is a big thing like it is*, *I definitely think [inhalation services] would be a good thing*.*”*

Overall, participants emphasized the importance of having inhalation services and suggested that there would be an increase in site uptake if the site offered such a service: *“I’d be here every day if they offered inhalation services*.*”*

*Rules regarding splitting and sharing*. Another unfavorable policy was the inability to ‘split’ or ‘share’ drugs with others while in the consumption room. Some participants described that they typically split or shared their drugs with other people for various reasons, including for safety when they do not want to use alone, or when pooling their money together to purchase a larger quantity of drugs than just one could afford, with the idea that they would consume the drugs together. Thus, participants suggested that because the site did not allow this practice, it was not reflective of their use patterns, and was a deterrent to using the consumption room. For example, one participant explained the current policy is not commensurate with how PWUD prefer to use their drugs:

*“Because you’d maybe want two people to go in*, *like a couple*. *I don’t know how that works…for sharing sometimes*, *because that’s what happens on the street…Yeah*, *not like anything dirty or anything*. *But yeah*. *That would be a thing*.*”*

*Location*. Another commonly reported issue with the site was the location. The Spot is physically located approximately 5 minutes’ drive or a 15-20-minute walk from Sudbury’s downtown core. Most participants (70%; n = 14) noted that the distance to travel to the site acted as a major barrier to accessing the site. Participants often referred to the site as “out of the way”, and many articulated that the site’s location was situated too far from where they purchased their drugs, which deterred them from utilizing the site. Participants emphasized that the site should be relocated to downtown Sudbury, which would encourage access and uptake of the site:

*“Preferably I’d like the location to be downtown because that’s where everyone hangs out and that’s where the drugs are*. *It doesn’t make sense to buy drugs down there and walk all the way up here when you can just shoot it up down there*. *A lot of people say the same thing*.*”*

Participants reiterated they typically need or want to consume their drugs as soon as they get them and are reluctant to travel or walk a long distance to the site. This was particularly relevant for participants who shared experiences of going through withdrawal or feeling ‘dope sick’, and described how during these moments, there is usually an urgency and need to consume their drugs, and that their focus is on not feeling sick as opposed to figuring out where and how they can use more safely. In these instances, many PWUD prioritized relieving their withdrawal symptoms and ensuring they do not feel sick, instead of travelling to the site.:

*“I guarantee you guys don’t get people here because of the location*. *I know downtown don’t want it down there and I understand that*. *But it’s gotta be down there I think*. *A lot more people would be using this facility if it were closer to the downtown core*. *Because what do you do*? *You wake up in the morning and want to do your fix*, *you’re gonna walk a half a mile first*? *No…Because it’s the first thing you wanna do when you wake up*. *You roll over*, *sometimes sick and the first thing you want to do is get un-sick*. *If I can walk three minutes compared to 20 minutes*, *I would be here*.*”*

However, nearly a third of participants (n = 6; 30%) noted that they did not mind the location as they either lived nearby, or they preferred that it was outside of the ‘drug scene’, where they were less likely to be recognized or further stigmatized for using the site. For instance, one participant described the importance of the discreetness of the site, and how it made them feel more comfortable and willing to visit it:

*“I work part-time and if they knew I was using I might not have that job*. *My buddy here isn’t a user*. *He knows I do*, *but I try to hide it from everyone*, *I really do*. *I’m not afraid of people seeing me here because it is farther away*, *right*? *So the location is good in a way*, *I never thought of it that way*.*”*

Similarly, participants discussed potential impacts of the physical infrastructure of the building, particularly in regard to it being physically located in a trailer in an open field. For instance, two participants raised concerns about the lack of privacy of the location, as there were no surrounding buildings or greenery around the trailer. They expressed that this exposure made them feel paranoid or uncomfortable leaving the site, as there was potential to be visible to the public as they could easily be spotted. This raised issues related to the lack of privacy of the location, as described by one participant: *“It’s open and there’s no sneaking around*. *For people who are trying to hide their addiction they’re not hiding jack shit around here*.*”*

However, a few participants (n = 4; 20%) described how they appreciated that The Spot was physically located on an open field, as they would have an easier time monitoring their surroundings: *“I think most people are paranoid so it’s better that [The Spot]is open*. *So they can see everything*, *and no one is hiding*.*”*

*Hours of operation*. Another concern that participants commonly discussed related to the limited hours of operation. Currently, the site is only open from 10:00am until 4:00pm, seven days a week. All participants suggested that the hours were not long enough and did not reflect the times they typically consumed their drugs. Participants indicated that the site should be open for a longer duration of time during the day to ensure it met their diverse needs:

*“And the hours*, *like I said*, *most people don’t wake up at 10am*. *They’re usually up at like 7am*. *I think they should be open earlier and later*. *What about the nightlife*? *People go out and start drinking and the next thing you know they wanna do a fix*. *I know everything cost money too*, *like the workers*. *I get that*. *But if it’s gonna be done*, *it’s gotta be done right*. *That’s my opinion*.*”*

Over a third (n = 7, 35%) of participants suggested it would be ideal for the site to remain open 24 hours a day to accommodate PWUD. For instance, when asked if there was anything that could be improved about the site, one participant stated: *“Longer hours*. *I don’t know if it would be possible to have it open 24 hours*, *you know*?*”*

Participants also specifically discussed the importance of having the site open earlier than 10:00am for a variety of reasons. For instance, another participant reiterated how PWUD typically wake up in withdrawal or feeling ‘dope sick’, and the first thing they need to do is consume their drugs in order to feel better: *“Yeah*, *well people start withdrawing first thing in the morning*, *super early*. *Most people want their shit by 8 o’clock*. *Just follow the dealers and the street girls*.*”*

Other justifications for suggesting the site should open earlier included capitalizing on when people attended the methadone clinic to get their medications, and how many city shelters forced individuals to vacate early in the morning. As such, participants described the importance of having a place to consume their drugs that was accessible at that time:

*“Well*, *I know a lot of people*, *if they’re homeless*, *they get kicked out*. *Like that one [shelter]*, *you’re out at 7*:*00am*. *And a lot of methadone clinics*, *mine’s open at 7*:*30 and the other is open at 9*:*00*. *So that’s two hours [before the site opens]*. *And they need it every day*. *That’s why there’s dealers down there at that time*. *Just waiting*.*”*

## Discussion

This qualitative study examined client experiences and perceptions regarding Sudbury’s first SCS. Data collected suggest that the site presents considerable benefits to PWUD in Sudbury. Participants discussed the importance of the site’s ability to potentially reduce public use, overall frequency of drug use, and stigma, as well as increase feelings of safety regarding drug use, particularly as it relates to overdose risk. Participants also provided thoughtful suggestions to improve the service, such as the site’s location, hours, and policies related to the lack of inhalation services and inability to split or share drugs.

The Spot utilization data further demonstrate a need for the sustainability of the site as there has been a relatively stable increase in uptake and utilization of the site since its inception. The number of consumptions and visits steadily increased month over month until around April, reaching a peak of 202 and 128 respectively. However, the site thereafter experienced a temporary drop in both visits and consumptions, with 118 consumptions and 79 visits in May and 114 consumptions and 77 visits in June. This was followed by a rapid increase in July and August 2023, to numbers surpassing those recorded in the initial months. The number of consumptions and visits in July of 2023 was 224 and 153 respectively and there was 241 consumptions and 201 visits in August of 2023. Research suggests that there may be seasonal impacts on substance use as drug initiation, overdose emergency room visits,police calls for overdoses, and overdoses themselves usually tend to spike in the summer months [16, 17]. In terms of new clients, it appears that this number peaked in December 2022, and has remained relatively consistent within the last five months, with a small reduction in May 2023. Possible explanations for this trend may be that PWUD who were acutely in need of the SCS may have signed up in the initial months of opening, or there is a need for continued awareness and education around the site. Notably, the site also reversed a total of 20 overdoses, underscoring its usefulness as a safe space for PWUD to consume their substances and be attended to in the event of an overdose. Given the disproportionate rates of overdoses and related harms that have occurred in Sudbury, the ability of the site to respond to overdoses is extremely important [3].

In addition to the site’s ability to reduce overdose-related harms during the ongoing overdose crisis, participants discussed the value of The Spot in its ability to potentially address issues related to public consumption and related community-level stigma. Although these sentiments were largely speculative in nature and any impact will likely be contingent on continued sustainability and uptake of the site, available evidence suggests that SCS can, in fact, reduce public stigma toward PWUD through the reduction of crime rates, drug use, and prevalence of discarded needles in public spaces [7, 8, 18]. Data further suggest that SCS can achieve dual goals of reducing public consumption and visibility of drug use while reducing stigmatizing attitudes against them [19]. Considering the evidence, The Spot has the potential to increase perceptions of community safety, which can ideally work to address stigmatization of drug use in Sudbury. This is important to note for other non-urban communities who may experience community-level stigma against PWUD which acts as a deterrent to implementing evidence-based interventions, such as SCS [20, 21]. Recognizing that there might be initial pushback from communities to implement harm reduction programs in their respective neighborhood, it has been demonstrated that emphasizing the importance of overdose prevention can help decrease negative perceptions regarding harm reduction services [22]. As such, stakeholders involved in the implementation of SCS should engage with the public and mobilize discussions around the importance of harm reduction services as a means to reduce visibility of drug use and littering of paraphernalia, as this has the potential to mitigate negative attitudes.

Despite these potential benefits, there were a number of suggestions for how the service could be improved. Participants described issues related to the site’s location and operational hours, and suggested that due to its current location, the site is not reaching its full potential. Evidence suggests that travel acts as a significant barrier for PWUD in accessing health and social services [23, 24], and previous research has emphasized the importance of location and ensuring that travel or distance does not influence one’s ability or decision to use at the site [25–28]. Studies have documented that when harm reduction sites are located far away from where PWUD commonly congregate or are in inconvenient locations, this can act as a significant deterrent for individuals to access the site, which subsequently results in low uptake [24]. Suggestions to relocate the site within the downtown area where people typically purchase and use their substances and extend the operational hours were provided by participants as this could increase accessibility and uptake. However, given there were a minority of participants who emphasized the importance of the discreetness of the site’s current location, this may not benefit all PWUD. Finding a suitable location that appeases both the public but also meets PWUD’ needs is an ongoing challenge. For instance, The Spot is located where it is currently sits due to difficulties finding a location for the site that community members and city council could both agree on. This was a particular challenge, with members of the CDS proposing twelve unsuccessful locations. Deliberations took many months and resulted in advocates opening an unsanctioned temporary site in the interim, where The Spot is currently situated [29]. A systematic review on SCS design suggests PWUD prefer SCS that are open 24 hours a day and seven days a week, and that are near areas with high levels of drug use, health facilities, emergency services, and public transportation [25]. Evidence has also suggested that, when possible, SCS should be implemented in existing harm reduction facilities that are already being utilized by PWUD. This is relevant for other remote or rural communities potentially considering implementing a SCS, which tend to be smaller in population size and where there is less anonymity in comparison to urban settings. Having a stand-alone SCS that is not integrated within other models of care can impact an individual’s decision to access the site given the potential for exposure and associated stigmatization if seen accessing the site. Therefore, having the site integrated within other existing services as a one-stop-shop for PWUD within Sudbury could facilitate connections and referrals to other services. In a study by Jackson et al., (2022), PWUD in Nova Scotia primarily believed a SCS should be located in the same place as their local needle exchange, indicating the importance of a centralized and easily accessible location [30]. Many participants also suggested that SCS should be mobile, which can also increase accessibility [30]. However, finding a suitable location that community members can agree on can also be a contentious issue, which may be particularly relevant for smaller more tight-knit communities, as was the case for The Spot. Balancing the importance of ensuring consumption services are locally accessible and integrated within other health and harm reduction facilities, as well as the concerns of the community, can be difficult, and underscores the importance of community education on the beneficial impacts of SCS.

Another major drawback noted by participants was the lack of inhalation services within the site. Participants identified that in addition to injecting their substances, they would also inhale, some of whom noted doing so as a harm reduction measure. Many participants within our study expressed perceptions that smoking is safer than injection, with a few reporting that they would engage in smoking their drugs first to test them prior to injecting. Routes of consumption have been associated with distinct risks [31]. For instance, injection drug use is associated with increased risks for contracting blood borne viruses and bacterial infections compared to inhalation. However, in terms of risks for overdose, evidence suggests that PWUD who smoke opioids are more likely to use alone than those who use other routes of administration, which considerably increases the risk of overdose [31, 32]. Moreover, more individuals have been dying from opioid poisoning via inhalation than injection in Canada, and there has been a significant shift from injection as the primary route of administration [33–35]. For instance, reports from Alberta, British Columbia, and Ontario suggest that more PWUD experience overdoses due to inhalation than injection drug use, demonstrating the necessity of these services [34–36]. The rise in inhalation-related overdoses combined with perceptions that inhalation is inherently safer route of consumption among PWUD underscores the importance of offering inhalation services to clients. However, most existing SCS do not have inhalation services, aside from a few that were implemented in select jurisdictions within British Columbia and one recent location in Ontario. Although it is no longer in operation, North America’s first inhalation site was located in Alberta, Canada, and had seen an overwhelming response within the first months of its opening [37]. With its inhalation rooms constantly in use, the site had to expand its hours of operation to 24/7 and add more smoking rooms to keep up with demand and long lineups [37]. These findings indicate the necessity of inhalation services, and that PWUD will use these services if given the option to do so [37]. To ensure that The Spot is meeting the needs of all drug users in Sudbury, participants emphasized the importance of inhalation services. Allowing PWUD to smoke their substances in a safe space would enhance clients’ safety as medical personnel would be there to intervene. Further exploration on how administrative and logistical issues can be addressed to support the incorporation of such a vital service into The Spot, and in other SCS provincially, is warranted.

The issues discussed among participants indicate the need for The Spot to be reflective of PWUD diverse drug use patterns, needs, and lifestyles, including recognizing the increasingly common route of administration via inhalation [38, 39]. It is imperative to recognize that needs are different for each person, and policies must reflect this to ensure that The Spot achieves its goals of reducing overdose and related risks. To continue to meet the needs of PWUD in the City of Sudbury, it is imperative for the site to remain operational. Given the temporary nature of the site, the results of this initial exploration into client’s experiences can be drawn on to substantiate the need for site permanency within Sudbury, as well as the potential to relocate and integrate it into existing harm reduction services downtown. The results can also be used as an example for other rural, remote, or northern communities seeking to implement a SCS. In Ontario, securing long-term provincial funding and site permanency can be an uphill battle due to the multiple levels of approval and applications required. Sites must seek federal approval as well as provincial approval, both of which have similar onerous application processes (e.g., the need for extensive and ongoing community consultations, securing an optimal site location, etc.). Additionally, in Ontario, there is a provincial cap on the number of sites they will fund which sits at 21, and sites vying for this funding must demonstrate they can provide comprehensive wrap-around supports and community and city council support. This complicates the ability for communities to find the funding and support for SCS [10]. However, alternatives exist, including ‘urgent public health need sites’ (i.e., colloquially referred to as overdose prevention sites), which are temporary and can be implemented in the interim drawing on municipal funding as communities gather the data and support needed to submit a formal application to the government. For instance, Timmins, another small Northern Ontario community implemented an urgent public health needs site while awaiting federal and provincial approval to operate a permanent site [40]. These temporary sites are becoming more common as communities attempt to mitigate the burden of the opioid crisis and the difficulties involved in implementing SCS. As the policy and political landscape of SCS evolves in conjunction with the opioid crisis—which has disproportionately impacted smaller and northern communities in Ontario—it will be important to monitor the impact that these sites have on communities long-term. This underscores the need for a longer-term evaluation of the site’s uptake and ability to respond to overdoses and increase public safety.

### Limitations

Due to the small window of time (i.e., two and a half months) that the site was open prior to our study, it is likely that the utilization data is an underrepresentation of The Spots’ potential impact. Given this short timeline and the nature of qualitative studies, some responses were speculative and varied depending on whether the participant was discussing their experiences thus far or were sharing their thoughts on the potential impacts the site may have on them and their community in the future. Responses to our questions were also subject to a number of biases inherent in self-report and qualitative data including self-selection, recall and response biases, social desirability bias, etc. Furthermore, our sample is small and experiences (including those provided which were hypothetical or speculative) cannot be generalized outside of the specific participants and setting.

## Conclusion

This initial qualitative exploration of client experiences with Sudbury’s first SCS suggests that the site offers many benefits to PWUD within the community and can reduce fatal overdoses, underscoring its utility as a key harm reduction service that can potentially reduce community-level stigmatization. However, participants described issues regarding site operational policies that currently hinder site uptake and may not achieve the desired impact of the program. Suggestions to improve the site included the addition of inhalation services, relocating it to downtown Sudbury where PWUD congregate, and extending the operational hours. The results of this early study demonstrate the value and need for site permanency.
